# Acute Cardiorenal Syndrome: Epidemiology, Pathophysiology, Assessment, and Treatment

**DOI:** 10.31083/j.rcm2402040

**Published:** 2023-02-02

**Authors:** Xi Peng, Hui-Ping Zhang

**Affiliations:** ^1^Department of Cardiology, Beijing Hospital, National Center of Gerontology; Institute of Geriatric Medicine, Chinese Academy of Medical Sciences and Peking Union Medical College, 100730 Beijing, China

**Keywords:** cardiorenal syndrome, acute kidney injury, acute heart failure, organ crosstalk

## Abstract

Acute cardiorenal syndrome (CRS) is often observed in patients with acute kidney injury (AKI) in the cardiac intensive care unit and is reported to be associated with poor prognosis. Volume disorder or re-distribution, renin-angiotensin-aldosterone system activation, and neurohormonal and sympathetic nervous system activation have been suggested to be related to the occurrence of acute CRS. There is a lack of biomarkers that can identify changes in renal function in patients with acute CRS. Evidence-based medications are limited in the management of acute CRS in AKI. Therefore, we reviewed the epidemiology, pathophysiology, clinical assessment, and treatment of acute CRS in AKI.

## 1. Introduction

The concept of cardiorenal syndrome (CRS) was proposed to emphasize the interaction between the heart and the kidney. Traditionally, CRS is classified into five types according to primary organ dysfunction and acute or chronic characteristics (Table 1) [1]. This CRS classification provided insight into the multidisciplinary management of cardiovascular and renal systems. Considering the specific pathophysiological mechanisms involved, it is important to distinguish acute CRS as a subset of acute kidney injury (AKI). Acute CRS is defined as an extreme form of cardiorenal dysregulation in which treatment of decongestion of acute heart failure (HF) is limited by further worsening of renal function [2]. In clinical practice, acute CRS includes CRS types 1 and 3, as well as part of CRS type 5 involving both secondary acute cardiac and renal dysfunction [3].

**Table 1.** **Classification of cardiorenal syndromes**.

	Syndromes	Definition
Type-1	Acute cardio-renal syndrome	Acute HF leading to AKI
Type-2	Chronic cardio-renal syndrome	Chronic HF leading to chronic kidney dysfunction
Type-3	Acute reno-cardiac syndrome	AKI leading to Acute HF
Type-4	Chronic reno-cardiac syndrome	Chronic kidney disease leading to chronic HF
Type-5	Secondary cardio-renal syndromes	Acute or chronic systemic conditions leading to simultaneous impairment of heart and kidney

HF, heart failure; AKI, acute kidney injury.

AKI is currently diagnosed using the KDIGO (The kidney disease improving global outcomes) criteria [4]. Some studies have suggested that the patient’s clinical status should be considered when performing a comprehensive evaluation of renal function in patients with acute HF (Table 2, Ref. [4]). However, some aspects of acute CRS remain unclear. For example, further study of novel biomarkers that can detect changes in renal function is warranted. Additionally, effective therapy for acute CRS in AKI is a noteworthy issue. In this review, we focus on acute CRS and the crosstalk between the heart and the kidney, exploring the epidemiology, pathophysiology, clinical assessment, and the treatment of acute CRS in AKI.

**Table 2.** **The commonly used criteria for diagnosing AKI**.

	Stage	Serum creatinine criteria	Urine output criteria	Suggested additional criteria
KDIGO	1	1.5–1.9 times baseline within 7 days OR ≥0.3 mg/dL (≥26.5 µmol/L) increase within 48 hours	<0.5 mL/kg/h for 6–12 hours	
	2	2.0–2.9 times baseline	<0.5 mL/kg/h for ≥12 hours	
	3	≥3.0 times baseline OR ≥4.0 mg/dL (≥353.6 µmol/L) increase OR initiation of renal replacement therapy	<0.3 mL/kg/h for ≥24 hours OR anuria ≥12 hours	
AKI in acute HF [4]		1.5–1.9 times baseline within 7 days before or during hospitalization OR ≥0.3 mg/dL (≥26.5 µmol/L) increase within 48 hours	<0.5 mL/kg/h for 6–12 hours	Deterioration in HF status or failure to improve OR need for inotropes, ultrafiltration or renal replacement therapy

HF, heart failure; AKI, acute kidney injury; KDIGO, the kidney disease improving global outcomes; OR, logical OR.

## 2. Epidemiology

Approximately one in five patients with acute heart disease has experienced AKI or worsening renal function (WRF) during the course of the disease and showed that AKI is associated with a five-fold increased risk for death and longer hospitalization [5]. In the cardiac intensive care unit, the reported prevalence of AKI is approximately 50% [5]. The strongest independent predictors of dialysis-requiring AKI (D-AKI) were mechanical ventilation (odds ratio [OR]: 7.06, 95% confidence interval [CI]: 6.69–7.45, *p *< 0.01) and sepsis (OR: 6.23, 95% CI: 5.86–6.64, *p *< 0.01). Other risk factors for D-AKI included hypertension (OR: 2.13), acute or chronic liver disease (OR: 2.01), diabetes mellitus (OR: 1.42), cardiac catheterizations (OR: 1.31), human immunodeficiency virus infection (OR: 1.31), and chronic kidney disease (OR: 1.14) [6]. In acute coronary syndrome (ACS), Killip class ≥3 and extensive anterior myocardial infarction are associated with a higher risk of AKI [7].

However, the clinical situations are more complicated. In patients with HF, treatment that results in a reduced glomerular filtration rate may represent a benign clinical change rather than a potentially harmful effect. The Diuretic Optimization Strategies Evaluation study showed that in patients with acute decompensated heart failure (ADHF), improvement in renal function was associated with a higher risk of death, rehospitalization, and emergency visits [8]. This implies that elevated serum creatinine levels do not adversely affect outcomes in all conditions. These data suggest that further studies are required to explain the association of acute changes in renal function with clinical outcomes.

## 3. Pathophysiology

**Fig. 1.**
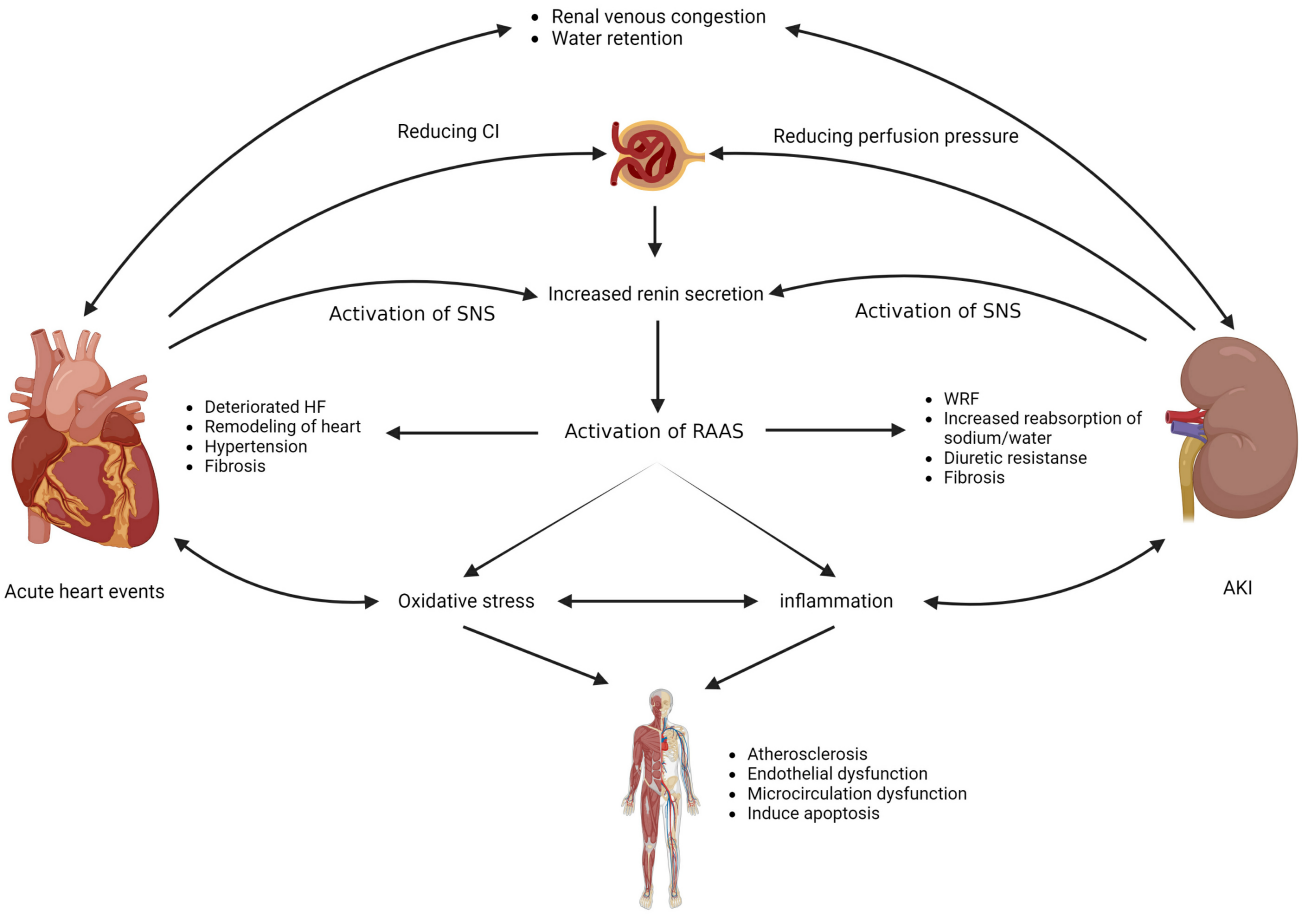
**Organ crosstalk between heart and kidney**. CI, cardiac index; SNS, sympathetic nervous system; RAAS, renin-angiotensin-aldosterone system; HF, heart failure; WRF, worsening renal function. Figure details inspired from Fu *et al*. [9], (Figure was created with BioRender.com).

In acute CRS, deteriorating cardiac and renal functions are not present in isolation but with mutual effects; therefore, the pathophysiological mechanism is multifaceted and complex (Fig. 1, Ref. [9]).

### 3.1 Hemodynamic

Initially, renal dysfunction in acute heart failure was considered to be caused by prerenal hypoperfusion due to a decrease in cardiac output. Inadequate renal blood flow or perfusion pressure leads to increased renin release by the juxtaglomerular cells of the afferent arterioles, which in turn triggers renin-angiotensin-aldosterone system (RAAS) activation, neurohormonal activation with sympathetic nervous system (SNS) activation, and non-osmotic vasopressin release. These factors lead to the retention of fluid, increased preload, deterioration of the renal system, and pump failure. It is difficult to justify the pathophysiology of acute CRS considering only the cardiac index reduction. The Evaluation Study of Congestive Heart Failure and Pulmonary Artery Catheterization Effectiveness trial found that poor forward flow alone did not account for the development of baseline renal insufficiency (RI) or WRF in these patients [10]. Nonetheless, a recent study found that decreased cardiac index was associated with a higher incidence of AKI [11]. This discrepancy may result from the different inclusion criteria of the two studies. It suggests that during a slight or moderate decline in cardiac index, the body can ensure renal perfusion pressure through mechanisms, such as neurohumoral regulation, while the body decompensates when the cardiac index drops severely, which leads to AKI.

Renal venous congestion is now increasingly recognized as an important hemodynamic mechanism of acute CRS. Blood flow through the kidneys is determined by the pressure gradient between glomerular capillaries. Chen *et al*. [12] showed that in those with isolated left ventricular dysfunction and biventricular dysfunction, AKI was associated with an approximately two-fold higher risk of hospital mortality; however, in those with isolated right ventricular dysfunction, AKI was associated with a 7.85-fold greater risk of death (95% CI: 2.89–21.3, *p *< 0.001). Additionally, in those with normal biventricular function, peripheral edema was associated with a higher adjusted risk of AKI (OR: 1.52, 95% CI: 1.07–2.45, *p* = 0.02). Peripheral edema, such as splanchnic and intestinal congestion, may cause increased intraabdominal pressure and renal vein pressure to worsen renal function.

### 3.2 Neurohormonal Dysregulation

Activation of SNS and RAAS occurs in acute CRS, which is a compensatory mechanism to maintain organ perfusion pressure. Activation of RAAS leads to an increase in angiotensin II, which leads to systemic vasoconstriction and expansion of extracellular volume by increasing sodium retention. However, a long-term increase in angiotensin II levels induces adverse effects on both the heart and the kidney. In the cardiac system, it increases myocardial oxygen demand and accelerates fibrosis, apoptotic processes, and myocyte hypertrophy [13]. In the kidney, activation of SNS and RAAS causes a reduction in the effective filtration rate by inducing efferent and afferent arterial vasoconstrictions. Angiotensin II increases sodium reabsorption through aldosterone action and promotes the generation of endothelin 1, which leads to inflammation and fibrosis in the kidney [13]. Theoretically, RAAS inhibitors are beneficial to patients with acute CRS; however, the appropriate and safe time to administer RAAS inhibitors in patients with unstable hemodynamics needs to be further confirmed.

### 3.3 Oxidative Stress and Inflammation

Oxidative stress and inflammation are interdependent, and their role in inducing cell damage, interstitial fibrosis, and organ dysfunction is well known. Overproduction of angiotensin II, sympathetic hyperactivation, ischemic injury, and venous congestion can increase free radical products, reactive oxygen species (ROS), and inflammatory cytokine. Oxidative stress causes interleukin (IL)-6, IL-1, and tumor necrosis factor-alpha release and hinders renal compensatory mechanisms [14]. After ischemia-reperfusion injury, neutrophils infiltrate into tissues and subsequently produce harmful proteases, ROS, and myeloperoxidase enzymes to impair organ function [14, 15]. Recent research suggests that IL-1β produced after renal ischemia-reperfusion may increase the risk of arrhythmias [16]. Hyperactive inflammation disrupts the cardiorenal crosstalk and results in adverse outcomes [17]. However, there is no recommended immunotherapy for interrupting the pro-inflammatory cytokine cascade in CRS.

## 4. Clinical Assessment

Volume overload and renal congestion are central to distinguishing acute CRS from other causes of AKI. Some biomarkers or imaging techniques have the potential to discern acute CRS.

### 4.1 Biomarkers

#### 4.1.1 Markers of Volume Assessment

Brain natriuretic peptide (BNP) and N-terminal prohormone of BNP, associated with ventricular filling pressure, are usually used to assess volume load. They vary according to sex, age, arrhythmia such as atrial fibrillation, and especially in renal dysfunction. Thus, they are limited in assessing the true volume status of patients with acute CRS. The plasma levels of carbohydrate antigen-125 (CA125) have recently been shown to be associated with the presence of congestive intrarenal venous flow in patients with acute heart failure, and it is not substantially influenced by age, weight, and kidney function [18]. Therefore, CA125 is a potential alternative indicator for guiding the intensity of diuretic therapy in patients with acute heart failure and kidney dysfunction [18].

#### 4.1.2 Markers of AKI

The 23rd Acute Disease Quality Initiative meeting recommended that biomarkers of AKI should be divided into stress, functional, and damage. These biomarkers can be used in combination with clinical conditions to identify high-risk patients, improve diagnostic accuracy of AKI, and assist in its management [19]. Among the indicators of stress, urinary Dickkopf-3, urinary metalloproteinases 2 and insulin-like growth factor binding protein 7 may be helpful in predicting the likelihood of developing AKI. Serum creatinine plays a pivotal role in evaluating renal function and is used to estimate glomerular filtration rate (eGFR). However, the level of creatinine often lags behind the clinical situation and tends to be largely influenced by muscle mass, activity, diet, and age. Functional markers such as plasma Cystatin C and Proenkephalin A are less reliant on muscle mass and dietary intake and can be used to assess GFR. Renal damage markers include urine C-C motif chemokine ligand 14, urine Interleukin-18, urine Kidney injury molecule-1, urine/plasma MicroRNA, urine N-acetyl-β-D-glucosaminidase, urine/plasma neutrophil gelatinase-associated lipocalin, etc. It is imperative that these biomarkers be evaluated in further detail in order to determine whether they can improve patient management alone or when combined.

Recent studies have also identified several biomarkers related to crosstalk between the heart and kidney [20]. Soluble suppression of tumorigenicity 2 (sST2), a member of the IL-1 receptor family, is released by cardiomyocytes and pulmonary endothelial cells and contributes distinctively to organ fibrosis [21]. Not only does sST2 present potential in predicting AKI and CRS in acute cardiac events, but IL-33/ST2 axis induces fibrosis which is appealing as an emerging mechanistic bio profile in CRS [21]. Similarly, tumor necrosis factor-like weak inducer of apoptosis (TWEAK) is a tumor necrosis factor superfamily member that activates the fibroblast growth factor-inducible-14 (Fn14) receptor to initiate multiple signaling cascades. The TWEAK-Fn14 axis may contribute to heart and kidney remodeling, including proliferation, inflammation and fibrosis, and blocking antibodies are being developed [22].

Angiopoietin-1, released by pericytes and platelets, is an agonist for the tyrosine kinase receptor (Tie-2), which exerts a protective effect by inhibiting vascular endothelial growth factor (VEGF) expression to stabilize the endothelium and prevent leakage from microcirculatory capillaries. On the contrary, angiopoietin-2 is an antagonist for Tie-2 and promotes endothelial permeability and inflammation. Besides, the soluble thrombomodulin (sTM) is also connected with endothelial injury. Plasma angiopoietin-2 and sTM levels are independent predictors of AKI in patients with acute myocardial infarction [23].

In addition, with the development of sequencing technologies, the pathogenesis of CRS at the molecular level is gaining attention. MicroRNA (miRNA)-21, a small non-coding RNA, is involved in several gene expression regulatory programs, and its elevated levels are associated with organ fibrosis [24]. miRNA-21 promotes the transition from quiescent fibroblasts to myofibroblasts by facilitating macrophage-fibroblast communication in a paracrine manner. The miRNA-21 promotes renal fibrosis by inhibiting Notch2 expression and contributes to the progression of acute kidney disease [25]. Thus, inhibiting miRNA-21 in macrophages is promising as a therapeutic target [24].

### 4.2 Imaging

#### 4.2.1 Sonography

Ultrasonic cardiogram not only reveals the situation of biventricular function, but is also affordable, repeatable, can be rapidly established, and guides diuretic therapy strategies [12]. Due to the recognition of the status of persistent systemic venous congestion in CRS, recently, renal venous impedance index and arterial resistive index evaluated by pulsed wave doppler sonography have shown potential value in acute CRS. A high arterial resistive index (OR: 6.25, 95% CI: 1.84–14.3, *p* = 0.003) may predict the improvement of serum creatinine levels with diuretic therapy in patients with type-1 CRS [26].

#### 4.2.2 Magnetic Resonance Imaging (MRI)

Microcirculatory dysfunction may play an important role in the pathogenesis of various types of CRS. Reinstadler *et al*. [27] used cardiac magnetic resonance to evaluate microvascular myocardial damage in patients with ST-elevation myocardial infarction treated by primary percutaneous coronary intervention and concluded that microvascular injury was the independent predictor of AKI (OR: 6.74, 95% CI: 1.49–30.43, *p* = 0.01). Furthermore, multiple parameters of MRI were used to assess CRS; for example, the diffusion-weighted imaging and T1 mapping techniques may contribute to the evaluation of AKI in acute CRS [28].

## 5. Treatment

Treating AKI generally requires discontinuation and avoidance of drugs that are harmful to renal function, such as antimicrobial drugs, non-steroidal anti-inflammatory drugs, and contrast medium. However, managing patients with acute CRS is challenging and dilemmatic.

### 5.1 Diuretics

The possibility that using diuretics may induce or aggravate WRF by inducing prerenal hypoperfusion limits the appropriate use of diuretics. Acute CRS is characterized by volume overload, and diuretics are the cornerstone of the treatment of acute CRS. WRF is not always associated with worse outcomes in patients with heart failure [8, 29].

#### 5.1.1 Diuretic Resistance

Diuretic resistance is a relative term, which implies the inability to decongest despite adequate and escalating doses of diuretics. Mechanisms of diuretic resistance have not been completely clarified, which may include multiple reasons. Hypoalbuminemia may impair the uptake and secretion of active loop diuretics and in the context of acute CRS, elevated levels of organic acids, such as blood urea nitrogen, can compete for loop diuretic entry into the nephron [30]. After a period of continuous administration, distal convoluted tubules are remodeled, which allows these sites to reabsorb sodium more efficiently, thereby creating resistance to diuretics [30]. Additionally, RAAS and SNS activation and some nephrotoxic or anti-natriuretic drugs, such as non-steroidal anti-inflammatory agents, are associated with the inefficiency of diuretics [31].

#### 5.1.2 Strategies to Overcome Diuretic Resistance

**Fig. 2.**
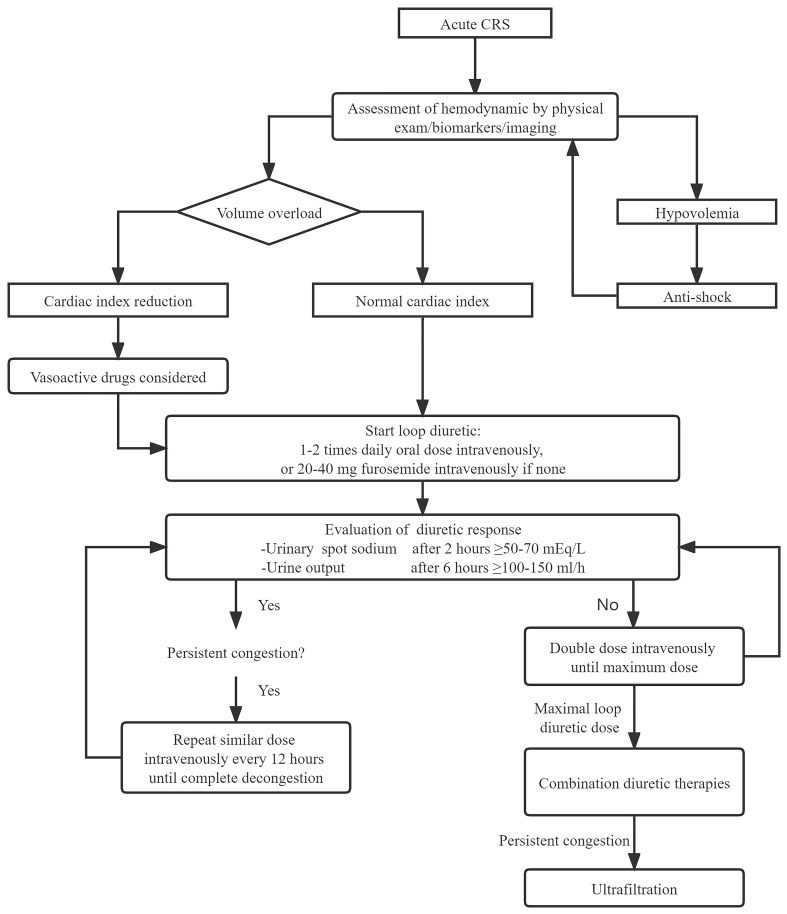
**Management of acute CRS, the part of diuretic response adapted from the 2021 ESC guidelines for heart failure [32]**. CRS, cardiorenal syndrome.

The European Society of Cardiology guidelines suggest an approach toward stepped pharmacologic diuretic strategies based on diuretic response (Fig. 2, Ref. [32]). When the required decongestion is not obtained with a high dose of loop diuretic monotherapy, combination diuretic therapy should be considered.

Acetazolamide is a classical and almost forgotten diuretic agent that boosts the effect of loop diuretics by inhibiting sodium bicarbonate reabsorption in the renal proximal tubules and offering more sodium to Henle’s loop. Acetazolamide also protects the nephron from ischemia-reperfusion injury by renal vasodilatory effect [33]. In a study on patients with ADHF at high risk for diuretic resistance, acetazolamide with low-dose loop diuretic resulted in similar natriuresis when compared with raising loop diuretic dosing alone [33].

Sodium overload has been considered to lead to the deterioration of ADHF. However, real-world analysis in patients with refractory ADHF showed that hypertonic saline (sodium chloride concentration >0.9%) along with loop diuretic administration was associated with increased diuretic efficiency and weight loss and improved renal function and metabolic derangements. Furthermore, hypertonic saline did not adversely result in respiratory or electrolyte balance [34]. Although it is a potential treatment, the mechanism is unclear, and the dose and safety of hypertonic saline need further investigation.

Finally, a high-dose loop diuretic combination with metolazone, tolvaptan, or intravenous chlorothiazide has also been shown to increase urine output in ADHF complicated by diuretic resistance, and there were no significant differences between the groups [35].

### 5.2 Neurohormonal Antagonists

Although inhibitors of the RAAS are known to improve the prognosis of heart failure, clinicians’ concern regarding inducing hyperkalemia or WRF limits their use in acute CRS. Actually, the modest decline in eGFR should not be alarming if the patient’s clinical status does not worsen. This phenomenon is commonly referred to as pseudo-WRF, and more importantly, in the initial use of RAAS inhibitors, increased creatinine may even be associated with better outcomes [4]. Since the benefits of these drugs outweigh the risks associated with WRF, they can be titrated based on close monitoring of potassium and renal functions [36].

### 5.3 Sodium-Glucose Cotransporter-2 (SGLT-2) Inhibitors

SGLT-2 inhibitors reduce glucose reabsorption by blocking the SGLT-2 of the proximal convoluted tubule while increasing natriuretic effect and reducing volume overload. As a class of glucose-lowering drugs, SGLT-2 inhibitors have excellent action in improving the prognosis of cardiovascular diseases. Notably, SGLT-2 inhibitors also significantly reduce the risk of progression of kidney disease. In type-2 diabetic rats, inhibition of SGLT-2 protected the kidney from myocardial infarction-induced CRS, possibly by reduction of renal oxidative stress [37]. Moreover, SGLT-2 inhibitors may reverse endothelial dysfunction to improve cardiomyocyte function in CRS. A dosage of 1-μM empagliflozin can reduce uremic serum-induced ROS levels by 63% [38]. It provides new information on the role of endothelial function in the crosstalk between the heart and the kidney [39].

### 5.4 Vasoactive Drugs

In theory, inotropic agents can increase cardiac output, improve renal blood flow, and improve right ventricular output, thereby relieving systemic congestion. However, no inotropes or vasodilators have been shown to improve outcomes in patients with CRS [40]. Even in studies that demonstrated increased urine output due to the administration of low-dose dobutamine or dopamine, there was no significant difference in changes in clinical outcomes [40].

Levosimendan exerts its inotropic effects by increasing troponin C-to-calcium sensitivity in cardiomyocytes via cyclic adenosine monophosphate effects. Furthermore, it has vasodilatory effects by acting on adenosine triphosphate-sensitive potassium channels in smooth muscle cells. Thus, in addition to improving left ventricular function, levosimendan may also induce preglomerular vasodilation and increased renal blood flow. In a randomized double-blind study in patients with HF and impaired renal function, the glomerular filtration rate increased by 22% in the levosimendan group compared with the dobutamine group (*p* = 0.012) [41]. The long-term safety of levosimendan has also been determined [42]; therefore, levosimendan could be the preferred inotropic agent for treatment in patients with low cardiac output-induced CRS [41, 42].

### 5.5 Ultrafiltration

Ultrafiltration is an effective strategy of decongestion, and in patients with severe diuretic resistance to volume overload. However, it should only be considered when optimal diuretic therapy and other medical management maneuvers have failed to provide adequate results. In the Cardiorenal Rescue Study in Acute Decompensated Heart Failure, ultrafiltration as the preferred treatment was associated with a significant increase in serum creatinine and a higher rate of adverse events compared with the stepped diuretic algorithm (72% vs. 57%, *p* = 0.03), and there is no significant difference in the effect of decongestion (*p* = 0.58) [43].

## 6. Conclusions

Acute CRS in AKI is not a rare phenomenon and is associated with a poor prognosis. It is noticeable that acute CRS should be regarded as a unique form of AKI. Volume status data and renal venous congestion are essential in most situations. Studies have demonstrated the interaction among cardiorenal and other organs in acute CRS, which may lead to multiple organ dysfunction induced by neurohormonal dysregulation with RAAS and SNS activation, as well as oxidative stress along with inflammation. The management of acute CRS is challenging. There is an urgent need for multidisciplinary cooperation to further investigate the deep pathophysiological mechanism, prevent acute CRS occurrence and develop effective therapy methods.
